# A Case of Catalepsy

**Published:** 1858-08

**Authors:** 


### *A Case of Catalepsy.* By Dr. G. Buchanan.

This case presents no new facts in elucidation of pathology or treatment, but it affords a well-marked example of a very rare affection.

"On the 28th of May, 1856, I was suddenly called to visit Mr. X——. Proceeding immediately to the house, I found his mother, a widow lady, in great distress. She said, 'My son is very ill; look at him, there he is,' pointing to a bed-room door. Having had no information as to the nature of the case, I was struck with surprise at what met my view. Standing in the door-way of the room indicated was a young man about thirty years of age, stout and well made. He had stripped himself of coat, waistcoat, necktie, and boots; and in that state he was planted with his feet slightly apart and holding the door handle with one hand. Going up close to him, I asked what was the matter, but he paid no attention to what I said, nor answered a word. I now perceived that he was in a strange state. His eyes were wide open, staring straight forward, wholly devoid of expression, motionless, and with the pupils dilated. On taking hold of his arm, I found it was cold and hard as marble, and on asking him to move and trying to take his hand, I found the muscles firm as iron, and clenched tight as in cadaveric rigidity. I felt every part of his body, the muscles of his legs, abdomen, neck, and arms, all were equally fixed, tight, hard, and cold. His face was pale and cold, the lips livid, the jaws fixed; perspiration was visible in drops on his forehead. The pulse was 80, of fair strength, but was apparently soft; this, however, I presently found was fallacious, the apparent softness being caused by the tension of the tendons of the supinator longus and flexor carpi radialis muscles, which bore off the fingers from pressing on the artery. The breathing was subdued, but regular, and was occasionally accompanied with slight stertor, being performed entirely through the nose.

"Being utterly astonished at such a strange appearance, I tried to disengage his hand, with the view of leading him to his bed, which was not far off. With the greatest exertion I succeeded in unclasping it from the door-handle, which I had no sooner done, than with the other he seized the door-post, and with the one now released, grasped my own. It closed on mine with the force of a vice, and the spasmodic clutch made me wince with pain, but by no effort could I release it. I remained writhing in the gripe of a marble statue, insensible alike of his own state and mine. At last, by use of great force, I got clear, and stood aside a little to recover my breath. When I had arrived at the house, I learned that Mr. R. Connell, surgeon, had been sent for at the first, and had seen the patient; and while I was engaged in the personal struggle above detailed, Mr. Connell was sent for. On his arrival we again examined the patient together, and leaving him standing in his statue condition, retired to consider the nature of the case. The prominent symptom was fixation of the muscles, and every medical man at that time having his attention turned to the trial of Palmer, it is not strange that the idea of strychnine poisoning should have suggested itself to both of us. We, therefore, a second time scrutinized all the symptoms, and as I was at that time engaged in assisting at some experiments on the effects of strychnine on inferior animals, we had little difficulty in coming to the conclusion that the idea first entertained was inadmissible. The attack was evidently of a cataleptic nature. Our first object was to get the patient into the recumbent posture, and for that purpose had a sofa prepared in another room, preferring it to a bed on account of the freer access of air. We now tried to lead the cataleptic into the other other room, but he was planted so firmly that we could scarcely move him. Disengaging his hand from the door-post, we pulled him forward, when he fell over perfectly rigid like a pillar. We pulled him along in this state, but had to place him on his feet again for fear of hurting his toes, which were catching on the carpet. We then tried to carry him, but on getting him nearly level, the knees bent and he slipped from our hands, again assuming the erect posture. After awhile the hands began to twitch a little, and on pulling him, we now got him forward a few steps, soon, however, becoming rigid again. By dint of pushing and pulling, we got him conveyed to the sofa, on which we had little difficulty in laying him. Warmth was immediately applied to his feet and over the stomach, and cold to the head by means of wet towels. In a short time the hardness of the limbs wore off, his face became warm, he became relaxed in all his muscles, and could swallow a few spoonfuls of water. There seemed to be no indication for special treatment, and we left shortly after, with orders to continue application, and give a cup of tea when he appeared able to swallow it.

"Two hours after, in company with Mr. Connell, I again visited him. All symptoms of rigidity had passed off; he was lying perfectly easy on the sofa breathing naturally, his face a little flushed, and perspiring. He spoke sensibly, and said he was pretty well, but felt tired and sore, evidently from the violence we were under the necessity of using to get him to move on our former visit. He assured us he had never had any fit of the kind before, and the only reason he could assign as cause for the present one was, that he had been very much fatigued that day in removing from another house, and had taken almost no food. When Mr. Connell saw him about an hour before my arrival, he found him standing in his bed-room in the half undressed state, with a strange wild look, and the appearance of being tipsy. We found, however, that he had taken no spirits that day, and was not in the habit of indulging. He could answer questions, but was very excitable and obstinate. He was induced to lie down, and had an opiate administered. He told us afterwards that, feeling uncomfortable in bed, he had arisen to undress, when the attack came on and fixed him in the position in which I found him.

"On visiting him next morning, he seemed quite well. He had slept, and taken breakfast with relish. He was advised to remain at home that day, and take some purgative medicine. The day after he was well, and at his usual avocations.

"On the 22d April, 1857, I was again summoned in haste to see Mr. X. On entering the house, I found him in nearly exactly the same state as on the former occasion. Mr. Connell had again been called in in the emergency, and I found that gentleman supporting the patient, who was standing, stripped of coat and boots, and with one hand clutching at the gas bracket, and by the weight of his body had partially twisted it from its place. The symptoms were precisely those of the former attack, and a description of one will serve for both. What rivited our attention most on both occasions, was the fixed and unchanging attitude, the perfect hardness of the muscles, the pallor of the countenance, the glaring and wild expression of the eyes, and the icy coldness of the skin. Altogether, it would be difficult to conceive a more appalling spectacle. Having seen him during the previous attack, Mr. Connell and I at once knew how to proceed, and using force more promptly than before, we soon had him prostrate on the sofa, although not without considerable exertion. We had most difficulty in getting him into the horizontal posture, for by the time we had dragged him to the sofa, the rigidity had partly gone off, and he was partially conscious. He, however, struggled violently against us, not in the way of attack, but of resisting all our efforts to make him lie down. Mr. Connell, therefore, grappled with him, while I seized his feet, and by a rapid jerk both were precipitated. A little exertion freed Mr. Connell from his grasp, and he then lay quiet and stiff. The same means used as on the former occasion—warmth to the feet and stomach, and cold to the head—were soon followed by entire relaxation, and when we returned in two hours, we found him sensible and able to swallow a cup of tea, but strangely excitable and ill at ease. Next morning all evidence of the seizure had passed off.

"In the present instance, searching for some cause of the fit, I learned that the patient is subject to slight bilious attacks, that at this time his digestive organs were somewhat out of order, and that, meeting an old friend, he had accompanied him to the theatre, where he remained till a late hour; that during the day he had been in a restless state, and being unable to take a proper quantity of food, was much worn out in the evening, just before he was seized. The circumstances were not very dissimilar from those in which he was attacked on the previous occasion, the exciting cause in both being excitement, fatigue, and want of food. On questioning him as to his feelings during the attack, he said that he saw us, and although he did not remember all that passed, he knew quite well that we wished him to lie down, but that he had no control over his limbs, and had no power to assist, and was unconscious of resisting our efforts. As to his state during the day, he felt tired and excitable, and being quite worn out when he came home, he had resolved to go to bed for a rest, and was in the act of undressing, when he suddenly lost the power of his limbs, and would have fallen, he thinks, if he had not caught hold of the gas pipe, and in that position he stiffened, and was unable to move from it.

"I have had opportunities of seeing this gentleman in his ordinary state of health during the interval of the attacks, and since the last one. He is of a nervous and excitable temperament, and has quite the appearance that one would expect in the subject of some nervous affection.

"Mr. Connell informs me that he has been called to see him on two other occasions, viz: the 3d of July, 1856, and the 10th of May, 1857. On both of these dates he found him in a very restless and troublesome state, very stubborn, and partially insensible, but with no spasmodic or cataleptic seizure. They both occurred after a day of unusual fatigue. This state passed off without any treatment except rest, quiet, and an opiate."—*Glasgow Medical Journal*, from *Ranking's Abstract of Med. Sciences.*

---

*Palpitation of the Heart.*—In one of the meetings of the Physico-medical Society at Würzburk, (vide *Würzburger Verhandlungen*, VIII, 2, 1857 —*Medic. Neuigk.*,) Prof. Kölliker communicated that he had found a remedy to relieve in certain cases morbid palpitation of the heart. Reasoning from the experimentally established influence of the severe and constantly-returning palpitation, to relieve it by deep inspirations and subsequent holding of the breath. The advice was followed by good effect, a few deep respirations and moderate holding of the breath sufficing to arrest the palpitation for one or two days. Prof. Bamberger remarked that the expansion of the lungs, causing them to overlap the heart more fully, might render the palpitation only less perceptible, without actually arresting it. To this Kölliker replied, that it was improbable, because after a few deep inspirations palpitation had ceased, which otherwise had lasted for hours.—*Medical and Surgical Reporter.*

---

*M. Chas. Robin.*—The cultivators of histology and microscopy will be happy to hear that modern researches respecting the intimate nature of normal tissue and pathological products have now, at the Academy of Medicine, for the first time, a representative, in the person of M. Charles Robin, the well-known author of works bearing upon the study of tissue and of the secrets revealed by the microscope. The new academician was elected on the 11th instant, by forty votes, his principal opponent, M. Ménière, obtaining twenty out of the 74 votes which were polled.—*Lancet, May* 22, 1858.

